# Pertussis Toxin Stimulates IL-17 Production in Response to *Bordetella pertussis* Infection in Mice

**DOI:** 10.1371/journal.pone.0007079

**Published:** 2009-09-17

**Authors:** Charlotte Andreasen, Daniel A. Powell, Nicholas H. Carbonetti

**Affiliations:** Department of Microbiology and Immunology, University of Maryland School of Medicine, Baltimore, Maryland, United States of America; Instituto Butantan, Brazil

## Abstract

In a mouse model of respiratory tract infection by *Bordetella pertussis*, bacteria multiply in the airways over the first week and are then cleared over the next 3–4 weeks by the host immune response. Pertussis toxin (PT), a virulence factor secreted exclusively by *B. pertussis*, promotes bacterial growth in the airways by suppression and modulation of host immune responses. By comparison of wild type and PT-deficient strains, we examined the role of PT in modulating airway cytokine and chemokine responses affecting neutrophil recruitment during *B. pertussis* infection in mice. We found that, despite early inhibition of neutrophil recruitment by PT, high numbers of neutrophils were recruited to the airways by 4 days post-infection with the wild type strain, but not with the PT-deficient strain, and that this correlated with upregulation of neutrophil-attracting chemokine gene expression. In addition, there was similar upregulation of genes expressing the cytokines IL-17A (IL-17), TNF-α and IFN-γ, indicating a mixed Th1/Th17 response. Expression of IL-6, a cytokine involved in Th17 induction, was upregulated earlier than the IL-17 response. We showed that PT, rather than bacterial numbers, was important for induction of these responses. Flow cytometric analysis revealed that the IL-17-producing cells were macrophages and neutrophils as well as T cells, and were present predominantly in the airways rather than the lung tissue. Antibody neutralization of IL-17 significantly reduced chemokine gene expression and neutrophil recruitment to the airways, but only modestly increased peak bacterial loads. These data indicate that PT stimulates inflammatory responses by induction of Th1- and Th17-associated cytokines, including IL-17, during *B. pertussis* infection in mice, but a role for IL-17 in protection against the infection remains to be established.

## Introduction

*Bordetella pertussis* is a Gram-negative bacterial pathogen that infects the human respiratory tract and causes an acute disease known as pertussis or whooping cough. The bacteria adhere to ciliated cells and proliferate within the upper and lower respiratory tract, and do not disseminate to other tissues [1]. A mouse model of respiratory tract infection by this pathogen has been widely used to study the roles of various virulence factors, the associated pathology, and the immune responses elicited by the infection. Although symptomatic disease (cough) and transmission are not observed in the mouse model, several characteristics of the human disease are present, such as bacterial multiplication and clearance, limitation of the infection to the respiratory tract, and increased severity of infection in infant mice [2], [3]. In addition, systemic effects of the disease observed in humans, such as leukocytosis and hypoglycemia, can be detected in infected mice [3]. These characteristics, as well as the availability of inbred mice and genetically altered immunodeficient strains, make the mouse model useful for the study of host immune responses to *B. pertussis* infection.

*B. pertussis* produces several virulence-associated factors that contribute to the ability of this pathogen to infect the respiratory tract and cause disease. Among these virulence factors are two secreted toxins, pertussis toxin (PT) and adenylate cyclase toxin (ACT), both of which have suppressive and modulatory effects on the immune response [1]. PT, which is produced exclusively by *B. pertussis*, is a six-subunit exotoxin of AB_5_ configuration that ADP-ribosylates the α subunit of G_i_ proteins in mammalian cells, thereby inhibiting many cell signaling events through G protein-coupled receptors. By comparing wild type and PT-deficient *B. pertussis* strains in the mouse model, our group has recently shown that PT contributes significantly to bacterial growth in the respiratory tract by effects on host cells of the airways, including inhibitory effects on the protective role of resident airway macrophages [4], [5], [6]. PT also inhibits early neutrophil influx to the airways after infection by suppressing the early production of the neutrophil-attracting chemokines KC, LIX and MIP-2 by airway macrophages and epithelial cells [7].However, this latter property may not contribute significantly to bacterial growth, at least in naïve mice, since neutrophil depletion did not increase bacterial loads, possibly because of the inhibitory activities of PT and ACT on neutrophils [8].

Several studies have shown that PT and ACT also modulate cytokine responses produced by immune cells and during infection in the mouse model. ACT can upregulate major histocompatibility complex class II and costimulatory molecules on dendritic cells, inducing a semi-mature state that induces IL-10 production and decreases proinflammatory cytokine production [9], [10], [11]. However, ACT may also have proinflammatory properties since it can induce cyclooxygenase-2 (COX-2) and interleukin 6 (IL-6) production by macrophages in vitro [12]. PT can inhibit production of tumor necrosis factor-alpha (TNF-α) and IL-6, as well as various chemokines, by mouse macrophages in response to LPS [8], [13], [14]. However, PT can also synergize with LPS to induce proinflammatory cytokine production by dendritic cells [15]. Therefore the modulatory activities of these toxins on cytokine production are complex and may depend on several variables, including host cell type and other bacterial factors. How these toxins modulate cytokine immune responses during infection by *B. pertussis* is even less clear.

Several recent studies on immunomodulation by *B. pertussis* have shown that the host immune response may be skewed towards expansion of a subset of T lymphocytes termed Th17 cells, which produce the cytokine IL-17A (hereafter referred to as IL-17) [16], despite earlier studies having indicated that *B. pertussis* infection promotes a Th1 immune response (based largely on IFN-γ production) [2], [17]. Incubation of human dendritic cells with *B. pertussis* induced ACT-dependent expression of IL-23, a cytokine important for the expansion and maintenance of Th17 cells, but not of the Th1-inducing cytokine IL-12 [18], [19]. *B. pertussis* infection or pertussis vaccination of mice also induced an IL-17 response that was dependent on TLR4 signaling and IL-1, and that contributed to protection against *B. pertussis* challenge [16], [20]. Th17 cells are considered important for the host immune response against extracellular pathogens in the airways [21], [22], but are also associated with chronic inflammation and autoimmunity [23], [24]. IL-17 induces the production of inflammatory chemokines and cytokines and contributes to neutrophil recruitment and activation of innate immune cells [25], [26]. IL-17 is mainly produced by T cells but it can also be produced by neutrophils [22], [27] and NK cells [28]. Whether IL-17 and the Th17-polarized immune response contribute to protection against *B. pertussis* infection is still unclear, although this response has been shown to play a role in protection against another Gram-negative bacterial respiratory pathogen, *Klebsiella pneumoniae* [29].

Recently we have shown that PT inhibits the recruitment of neutrophils to *B. pertussis*-infected airways in mice over the first 2 days post-inoculation, but that this inhibition is subsequently overcome, with high numbers of neutrophils present in the airways by day 4 [5], [7], [8]. In this study we examined a more extended time course of neutrophil recruitment to the airways as well as cytokine and chemokine induction after *B. pertussis* infection, and tested the hypothesis that the later neutrophil recruitment is due to PT-dependent induction of a Th17 response and IL-17 activity.

## Results

### Effect of PT on kinetics of neutrophil recruitment to the airways in response to *B. pertussis* infection

**Figure 1:**
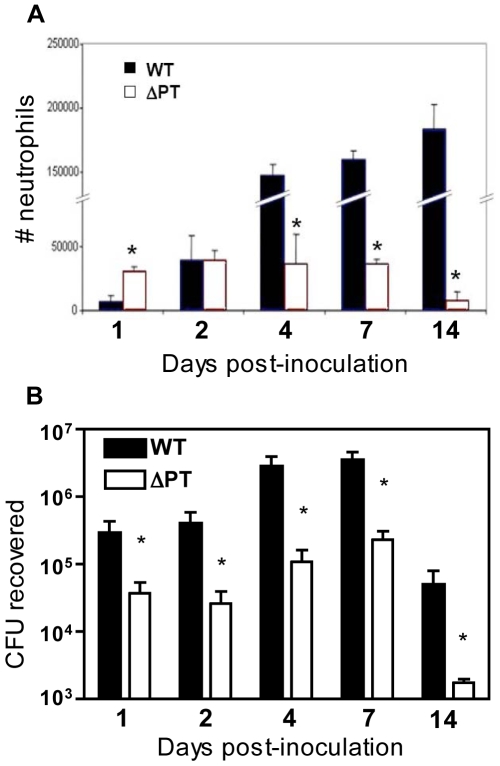
Kinetics of neutrophil recruitment during *B. pertussis* WT and ΔPT infection. (A) Numbers of neutrophils in the airways and (B) bacterial loads in the respiratory tract of mice at the indicated times post-infection with 5×10^5^ CFU of WT (closed bars) or ΔPT (open bars) strains. *n* = 4 mice/treatment group. The data represent 1 of 3 separate experiments with similar results. * Significantly different from WT (P<0.05).

Previously, by comparison of WT and ΔPT strains of *B. pertussis* we found that PT inhibits the recruitment of neutrophils to *B. pertussis*-infected airways in mice over the first 1–2 days post-inoculation, but that this inhibition is subsequently overcome, with high numbers of neutrophils present in the airways of WT-infected mice by day 4 [5], [30]. To determine the extended kinetics of neutrophil recruitment to the airways in response to WT and ΔPT infection, we inoculated mice with 5×10^5^ CFU of either strain (a dose at which ΔPT has a significant infection defect) and performed bronchoalveolar lavage (BAL) on groups of mice at several time points post-inoculation to determine the number of neutrophils present in the airways, as well as determining bacterial loads in the respiratory tract. As seen previously, early (day 1) recruitment of neutrophils to the airways was significantly lower (*p*<0.002) in response to WT than to ΔPT (Fig. 1A), but by day 2 post-inoculation, the difference in neutrophil recruitment in response to the two strains was eliminated (*p* = 0.64). On days 4, 7, and 14 post-inoculation, we saw high numbers of airway neutrophils in response to WT infection but not to ΔPT infection, with the numbers still rising at day 14 (Fig. 1A). This second wave of neutrophil recruitment in response to the WT strain occurred during the peak phase of infection, and lasted at least until day 14 post-inoculation when bacterial numbers were declining (Fig. 1B). As we have seen previously, bacterial loads of the WT strain were at least 10-fold higher than those of ΔPT at these time points (Fig. 1B), and so it is possible that high levels of bacterial factors, including LPS, can overcome the inhibitory effect of PT and stimulate the high level neutrophil recruitment in response to WT at the peak of infection. However, another possibility is that PT has a direct effect on this stimulation by modulating immune responses, thereby inducing a strong neutrophil recruitment in response to infection.

### Induction of lung chemokine gene expression at the peak of infection

**Figure 2:**
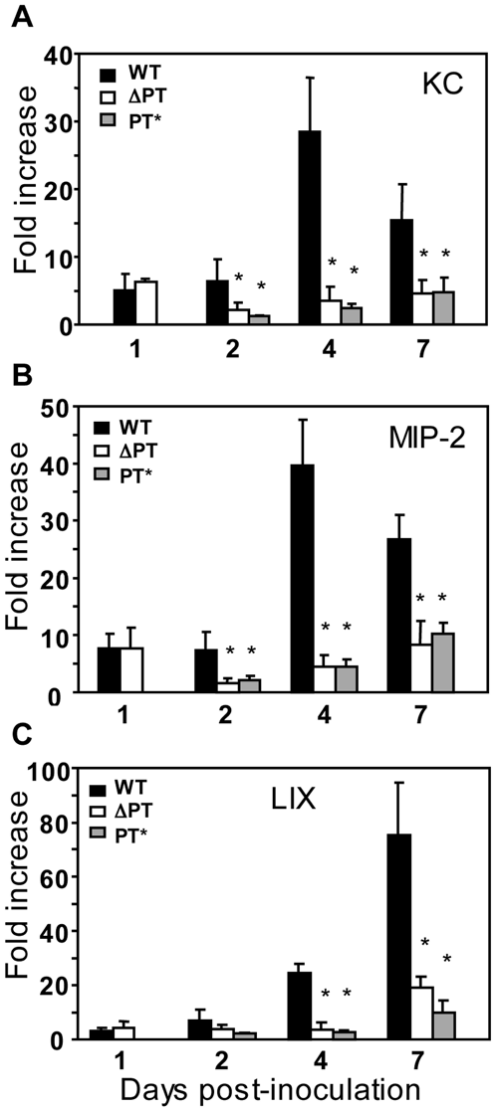
Kinetics of chemokine gene expression during *B. pertussis* WT, ΔPT and PT* infection. Fold increases in gene expression (relative to mock-infected mice) of the chemokines (A) KC, (B) MIP-2, and (C) LIX in lungs of mice at the indicated times post-infection with 5×10^5^ CFU of WT (black bars), ΔPT (open bars) or PT* (grey bars) strains. *n* = 4 mice/treatment group. The data represent 1 of 2 separate experiments with similar results. * Significantly different from WT (P<0.05).

To determine if the second wave of neutrophil recruitment in response to WT infection was due to induction of neutrophil-attracting chemokines, we analyzed the gene expression of KC, LIX and MIP-2 (ELR+ CXC chemokines that are important in neutrophil recruitment in mice and are functional homologues of IL-8 in humans) in lung samples on days 1, 2, 4 and 7 post-inoculation with 5×10^5^ CFU of either WT or ΔPT strains. Previously we found that expression of these chemokine genes was significantly increased above background at 6 h post-inoculation, that this early increase was significantly inhibited by PT, and that gene expression levels returned to background by 12 h post-inoculation [7]. This resulted in a higher level of neutrophil influx to the ΔPT strain than to the WT strain at day 1 post-inoculation (Fig. 1A and ref. 1). Though this early response is inhibited by PT, in contrast we observed strong induction of expression of all three chemokine genes in response to WT infection at later time points (Fig. 2). By day 2 post-inoculation, expression of KC was significantly higher in response to infection with WT than with ΔPT and at day 4, KC gene expression in response to WT infection was induced 28-fold above the control (mock-infected mice) and 8-fold above the level in response to ΔPT infection (Fig. 2A). The level of KC gene expression in response to WT was somewhat reduced by day 7 post-inoculation, although still significantly higher than the expression in response to ΔPT infection (Fig. 2A). A very similar expression profile was observed for MIP-2 gene expression in response to WT and ΔPT infection (Fig. 2B). LIX gene expression was also strongly induced in response to WT infection (Fig. 2C). At day 4 post-inoculation, there was a 20-fold increase in response to WT (above controls) and by day 7 post-inoculation, LIX was expressed 80-fold higher than controls, and at each of these time points LIX gene expression was significantly higher (4-6-fold) in response to WT than to ΔPT infection (Fig. 2C). We also determined the level of chemokine gene expression in mice infected with the *B. pertussis* PT* strain (that produces an enzymatically inactive form of PT) at days 2, 4 and 7 post-inoculation. The expression of chemokine genes in response to the PT* strain followed a very similar pattern to that of ΔPT (Fig. 2A–C), indicating that any differences in the responses to WT and ΔPT are due to the enzymatic activity of PT. Bacterial loads of the PT* strain were not significantly different from those of the ΔPT strain at the corresponding time points (data not shown), as we have previously observed [5].

### Expression of lung cytokines in response to WT and ΔPT infection

**Figure 3:**
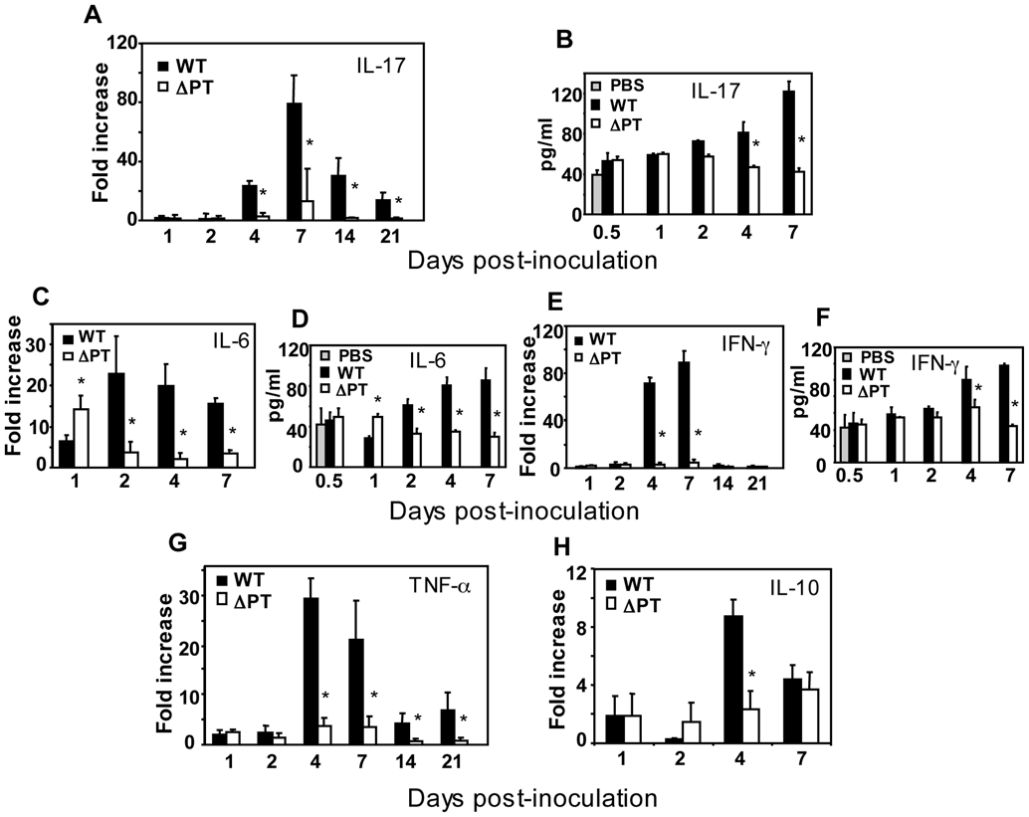
Kinetics of cytokine expression during *B. pertussis* WT and ΔPT infection. Fold increases in gene expression (relative to mock-infected mice) of the cytokines (A) IL-17, (C) IL-6, (E) IFN-γ, (G) TNF-α, and (H) IL-10, and corresponding protein levels of (B) IL-17, (D) IL-6 and (F) IFN-γ in lungs of mice at the indicated times post-infection with 5×10^5^ CFU of WT (black bars) or ΔPT (open bars) strains. *n* = 4 mice/treatment group. The data represent 1 of 2 separate experiments with similar results. *Significantly different from WT (P<0.05).

To investigate how the increase in chemokines and neutrophil recruitment in response to the WT strain at the peak of infection might be induced, we analyzed the expression of several other pro-inflammatory cytokine genes in the lungs in response to WT and ΔPT infection. The production of IL-17 is associated with induction of neutrophil-recruiting chemokines and subsequent neutrophil recruitment [24], suggesting a possible role for IL-17 in recruitment of neutrophils in response to *B. pertussis* at the peak of infection. Lung samples from mice infected with WT or ΔPT were analyzed for the expression of IL-17 to determine if there was a difference in the induction of this inflammatory cytokine in response to the two strains. IL-17 gene expression was not induced above control levels in response to WT or ΔPT over the first two days post-infection (Fig. 3A). By day 4 after WT infection, IL-17 gene expression was increased 20-fold above that of control and ΔPT groups, with a peak of approximately 80-fold induction on day 7 post-infection. While declining after day 7, IL-17 gene expression remained 15-25-fold higher in response to WT compared to that of control or ΔPT groups at least until day 20 after inoculation (Fig. 3A), despite low bacterial loads at these time points. At this dose (5×10^5^ CFU), we saw no significant increase in IL-17 gene expression in response to ΔPT over the control group. IL-17 protein expression in lungs was also significantly higher in WT-infected mice than in ΔPT-infected mice at the peak of infection (Fig. 3B). These data demonstrate that the kinetics of IL-17 expression closely matches that of chemokine upregulation and neutrophil recruitment to the airways, suggesting an association between these events, and that WT infection stimulates a prolonged upregulation of IL-17 gene expression.

TNF-α is another pro-inflammatory cytokine associated with Th17 responses, and IL-17 can further increase the production of TNF-α [23]. Low levels of TNF-α gene expression were detected in response to WT and ΔPT through day 2 post-infection, but at day 4 we observed a 30-fold increase in TNF-α gene expression in response to WT infection (Fig. 3G). TNF-α gene expression responses to ΔPT infection remained near background levels throughout (Fig. 3G). By days 14 and 20, TNF-α gene expression in response to WT infection was reduced to 5-7-fold above those of control and ΔPT groups, indicating an extended response to WT infection similar to that seen for IL-17.

IL-6 is a proinflammatory cytokine produced by several cell types and implicated in the induction of Th17 responses [24]. By 24 h post-infection, IL-6 gene expression in response to WT and ΔPT was 6- and 14-fold higher than that of the control group, respectively, suggesting an inhibitory role for PT on IL-6 gene expression early after infection (Fig. 3C). Subsequently, however, there was a 15-25-fold induction of IL-6 gene expression in response to WT infection, while the response to ΔPT was reduced to near background levels, indicating that PT may be responsible for IL-6 induction subsequent to the early inhibition. IL-6 protein levels reflected the differences in gene expression in response to infection with WT and ΔPT (Fig. 3D). The induction of IL-6 expression on day 2 after WT infection precedes the induction of IL-17 gene expression (not observed until day 4 – Fig. 3A), consistent with a possible role for IL-6 in inducing the IL-17 response.

IFN-γ is a cytokine typically associated with Th1 responses, but a mixed Th1/Th17 response to *B. pertussis* infection has previously been described [16]. In our study, no significant increases in IFN-γ gene expression or protein levels were observed in response to WT or ΔPT through day 2 post-infection (Fig. 3E,F). By day 4, however, there was a 70-fold increase in IFN-γ gene expression in response to WT infection, with a peak on day 7 post-infection (90-fold above control), whereas IFN-γ gene expression in response to ΔPT infection remained near control levels (Fig. 3E). IFN-γ protein levels were also significantly higher in response to infection with WT than with ΔPT at these time points (Fig. 3F). Unlike IL-17 and TNF-α gene expression, the IFN-γ response returned to control levels by day 14 post-infection (Fig. 3E), suggesting that a Th1 response is induced only at the peak of infection and is more transient than the Th17 response. These data indicate that *B. pertussis* infection induces a Th1/Th17 immune response in a mouse model. However, while the Th1 response appears to be relatively short-lived and IFN-γ is produced only during the peak of infection, the Th17 response is more prolonged and Th17 cytokines are elevated even while the bacteria are being cleared.

IL-10 is generally considered to be a Th2-associated cytokine and has immunosuppressive properties [31]. No significant increases in IL-10 gene expression were observed in response to WT or ΔPT through day 2 post-infection (Fig. 3H). By day 4, however, there was a 9-fold increase in IL-10 gene expression in response to WT infection, whereas IL-10 gene expression in response to ΔPT infection remained near control levels (Fig. 3H). At day 7 post-infection there was a 4-fold increase in IL-10 gene expression in response to both strains (Fig. 3H).

### The role of PT in induction of proinflammatory responses during *B. pertussis* infection

**Figure 4:**
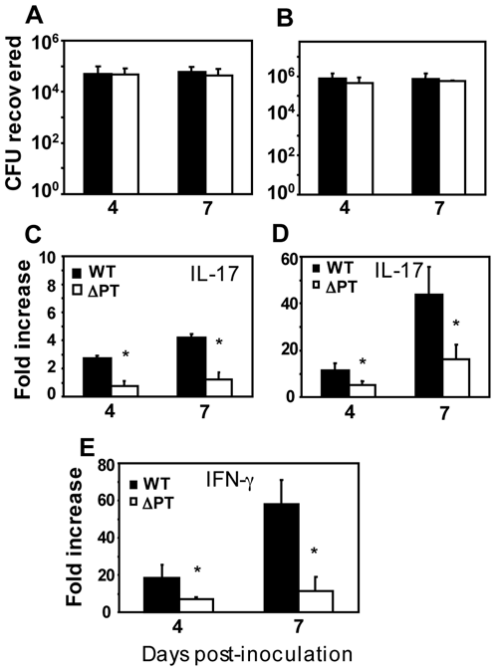
Effect of equalizing bacterial loads of WT and ΔPT on IL-17 expression. (A, B) Bacterial loads in the respiratory tract of mice on the indicated days post-infection with (A) 5×10^4^ CFU of WT (black bars) versus 5×10^5^ CFU of ΔPT (open bars), or (B) 5×10^5^ CFU of WT versus 5×10^6^ CFU of ΔPT (compare to difference in bacterial loads when inoculum doses are equal in Fig. 1). (C–E) Fold increases in gene expression (relative to mock-infected mice) of the cytokines IL-17 (C, D) or IFN-γ (E) in lungs of mice on the indicated days post-infection with the lower doses (C) or higher doses (D, E) of WT (black bars) or ΔPT (open bars) strains. *n* = 4 mice/treatment group. *Significantly different from WT (P<0.05).

As shown above, inoculation of mice with 5×10^5^ CFU of WT *B. pertussis* induces a strong Th1/Th17 response at the peak of infection, whereas infection with the same dose of ΔPT induces only low levels of inflammatory cytokines. Since the WT strain grows to significantly higher numbers in the airways than the ΔPT strain after day 2 post-infection (Fig. 1B), the increased inflammatory response to infection by the WT strain may be caused either by high bacterial loads or through a more direct induction by PT (or possibly both). In an attempt to discriminate between these two possibilities, we equalized bacterial loads of the 2 strains at the peak of infection by inoculating groups of mice with 10-fold higher levels of ΔPT than WT, which resulted in approximately equal bacterial loads in the airways on days 4 and 7 post-infection (Fig. 4A,B). We inoculated groups of mice with a low dose of WT (5×10^4^ CFU) versus our normal dose of ΔPT (5×10^5^ CFU) (Fig. 4A), or with the normal dose of WT (5×10^5^ CFU) versus a high dose of ΔPT (5×10^6^ CFU) (Fig. 4B), to examine the role of PT in induction of pro-inflammatory responses at different levels of infection. With the low dose of WT versus the regular dose of ΔPT, IL-17 gene expression showed a 3-4-fold induction in response to WT, but not to ΔPT, on days 4 and 7 after inoculation (Fig. 4C). Inoculation with the regular dose of WT versus a high dose of ΔPT resulted in strong induction of IL-17 gene expression in response to WT (10-fold on day 4 and 40-fold on day 7 - Fig. 4D), similar to that seen previously (Fig. 3A). Once again IL-17 gene expression was significantly lower in response to ΔPT infection (5-fold on day 4 and 15-fold on day 7 - Fig. 4D). Collectively these data indicate that PT makes a major contribution to induction of the IL-17 response during *B. pertussis* infection independently of bacterial load, but that induction of this response is not absolutely dependent upon PT, since infection with the higher dose of ΔPT resulted in IL-17 gene expression significantly above background.

To determine if the Th1 response to WT infection is also induced by PT, we examined IFN-γ gene expression, using the samples from mice infected with 5×10^5^ CFU of WT versus 5×10^6^ CFU of ΔPT. In response to WT infection, there was a 20-fold increase above controls in IFN-γ gene expression on day 4 and a 60-fold increase by day 7, significantly higher than that in response to ΔPT infection (5-10-fold increase above control) (Fig. 4E), indicating that PT induces Th1 as well as Th17 responses during *B. pertussis* infection.

### Flow cytometry analysis of IL-17-producing cells in mice infected with *B. pertussis* WT and ΔPT

**Figure 5:**
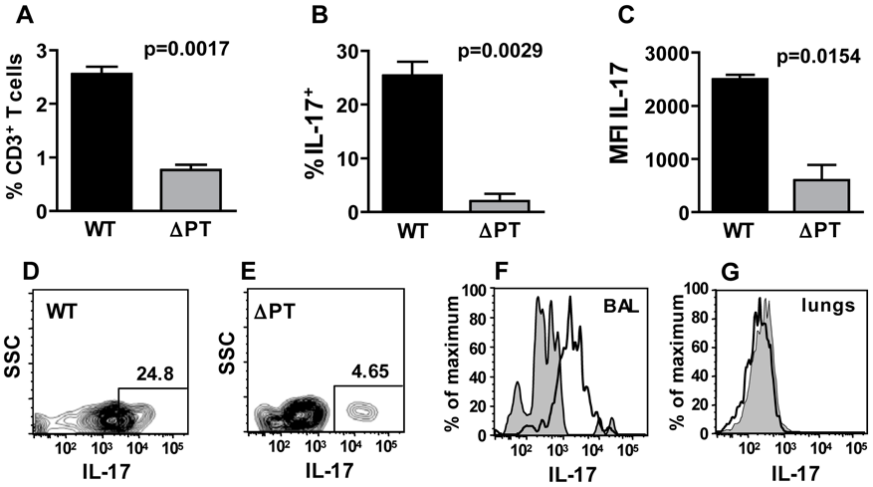
Flow cytometry analysis of airway T cells at the peak of WT or ΔPT infection. (A) Percentage of T cells (defined as CD3^+^) in the BAL fluid from mice infected with 5×10^5^ CFU of *B. pertussis* WT (black bars) or ΔPT (grey bars). (B, C) Percentage of T cells that stained positive for IL-17 (B) and mean fluorescence intensity (MFI) of IL-17 staining in those cells (C) in the BAL fluid of mice infected with 5×10^5^ CFU of *B. pertussis* WT (black bars) or ΔPT (grey bars). (D, E) Representative contour plots of IL-17 staining in T cells in the BAL fluid of mice infected with either WT or ΔPT. Number in box denotes the percentage of IL-17^+^ T cells. SSC – side scatter. (F, G) Representative histograms of IL-17 staining of T cells in the BAL fluid (F) or lung tissue (G) of mice infected with either WT (open plot) or ΔPT (shaded plot).

**Figure 6:**
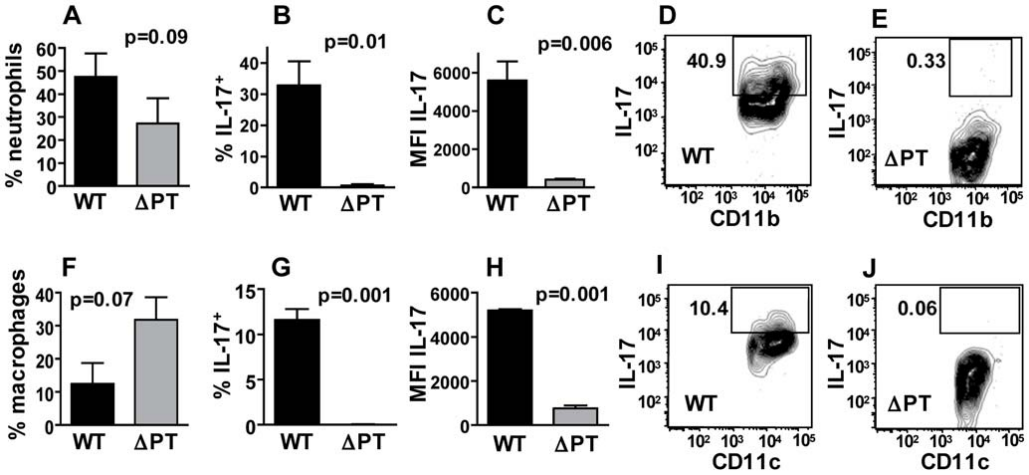
Flow cytometry analysis of airway neutrophils and macrophages at peak WT or ΔPT infection. (A) Percentage of neutrophils (defined as Gr1^hi^ CD11b^+^) in the BAL fluid from mice infected with 5×10^5^ CFU of *B. pertussis* WT (black bars) or ΔPT (grey bars). (B, C) Percentage of neutrophils that stained positive for IL-17 (B) and mean fluorescence intensity (MFI) of IL-17 staining in those cells (C) in the BAL fluid of mice infected with 5×10^5^ CFU of *B. pertussis* WT (black bars) or ΔPT (grey bars). (D, E) Representative contour plots of IL-17 versus CD11b staining in neutrophils in the BAL fluid of mice infected with either WT (D) or ΔPT (E). Number in box denotes the percentage of IL-17^+^ neutrophils. (F) Percentage of macrophages (defined as CD11b^−^ CD11c^+^ Gr1^lo^) in the BAL fluid from mice infected with 5×10^5^ CFU of *B. pertussis* WT (black bars) or ΔPT (grey bars). (G, H) Percentage of macrophages that stained positive for IL-17 (G) and MFI of IL-17 staining in those cells (H) in the BAL fluid of mice infected with 5×10^5^ CFU of *B. pertussis* WT (black bars) or ΔPT (grey bars). (I, J) Representative contour plots of IL-17 versus CD11c staining in macrophages in the BAL fluid of mice infected with either WT (I) or ΔPT (J). Number in box denotes the percentage of IL-17^+^ macrophages.

To confirm the difference in IL-17 production in WT- and ΔPT-infected mice and to identify the cells producing IL-17 at the peak of infection, we performed flow cytometry analysis of cells infiltrating the lungs and airways in response to these infections. Mice were infected with 5×10^5^ CFU of either strain, and lung and BAL cells from groups of mice (n = 3) were analyzed 7 days post-infection. CD3^+^ T cells made up 2.5% of BAL cells in WT-infected mice and 25% of these T cells were IL-17^+^, whereas less than 1% of BAL cells in ΔPT-infected mice were CD3^+^ T cells and only 2% of these were IL-17^+^ (Fig. 5A, B, D, E). In addition, the mean fluorescence intensity (MFI) of IL17 staining on CD3^+^ T cells among BAL cells from WT-infected mice was 5-fold higher than that from ΔPT-infected mice (Fig. 5C–E). Surprisingly, there was no significant presence of IL-17^+^ T cells in the lung tissue of WT-infected mice, and all of these cells appeared to be present in the airways (BAL cells - Fig. 5F, G). In addition, T cells were not the only producers of IL-17. Neutrophils (defined as Gr1^hi^ CD11b^+^) made up a greater percentage of BAL cells in WT-infected mice than in ΔPT-infected mice (Fig. 6A) consistent with our earlier data, and 33% of these neutrophils from WT-infected mice were IL-17^+^, whereas there was a very small number of weakly-staining IL-17^+^ neutrophils from ΔPT-infected mice (Fig. 6B–E), indicating that neutrophils are also significant IL-17 producers in this infection. Furthermore, 12% of airway macrophages (defined as CD11b^−^ CD11c^+^ Gr1^lo^) also stained positive for IL-17 in WT-infected mice, but less than 1% in ΔPT-infected mice (Fig. 6G–J). Again there were very few IL-17^+^ neutrophils or macrophages in the lung tissue of these mice (data not shown). Collectively these data confirm that the IL-17 response is significantly higher in WT-infected mice than in ΔPT-infected mice, supporting a role for PT in this induction, and demonstrate that multiple cell types (including T cells, neutrophils and macrophages) in the airways produce IL-17 at the peak of this infection.

### Neutrophil recruitment and chemokine gene expression after administration of α-IL-17 antibody

**Figure 7:**
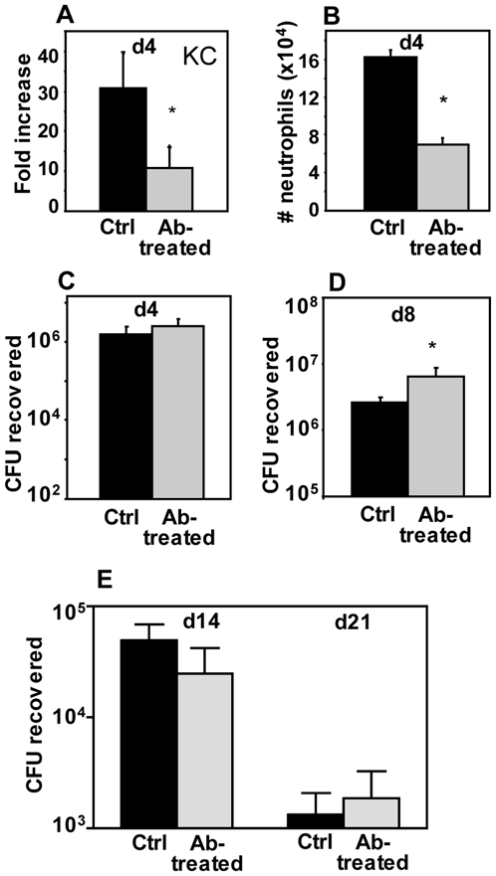
Effect of α–IL-17 antibody treatment on *B. pertussis* WT infection. (A–C) Fold increases in gene expression (relative to mock-infected mice) of KC (A), numbers of airway neutrophils (B), and bacterial loads in the respiratory tract (C) in control or α–IL-17 antibody-treated mice on day 4 post-infection with 5×10^5^ CFU of WT *B. pertussis*. Mice were treated with antibody intranasally on day 3 post-infection. (D) Bacterial loads in the respiratory tract of control or α–IL-17 antibody-treated mice on day 8 post-infection with 5×10^5^ CFU of WT *B. pertussis*. Mice were treated with antibody intranasally on days 3, 5 and 7 post-infection. (E) Bacterial loads in the respiratory tract of control or α–IL-17 antibody-treated mice on days 14 and 21 post-infection with 5×10^5^ CFU of WT *B. pertussis*. Mice were treated with antibody intranasally on days 3, 6, 9, 12, 15 and 18 post-infection. *Significantly different from control mice (P<0.05).

Th17 responses and the production of IL-17 have been associated with the induction of chemokine production and strong neutrophil recruitment [24]. To examine if the increase in chemokine gene expression and neutrophil recruitment in response to the WT strain during the peak phase of infection is induced by the production of IL-17, we administered 100 µg of α-IL-17 monoclonal antibody (or rat IgG to control mice) intranasally to mice on day 3 post-inoculation with 5×10^5^ CFU of the WT strain (a time point just prior to the observed increase in IL-17 expression – Fig. 3A). Groups of mice were euthanized on day 4 post-inoculation and KC gene expression, neutrophil recruitment and bacterial loads in the lungs were examined. KC gene expression in response to WT infection was induced approximately 30-fold above background levels (Fig. 7A), comparable to levels we observed previously in response to WT on day 4 post-infection (Fig. 2A). Treatment of mice with α-IL-17 monoclonal antibody significantly reduced KC expression in response to infection (Fig. 7A) and this corresponded to a significant reduction in neutrophil recruitment to the airways (Fig. 7B), indicating that IL-17 plays a role in inducing chemokine expression and subsequent neutrophil recruitment in response to WT *B. pertussis* at the peak of infection. Bacterial loads in these mice treated with α-IL-17 were 2-fold higher than those in control antibody-treated mice (Fig. 7C), but this difference was not statistically significant. To determine whether continued neutralization of IL-17 throughout the peak of infection would significantly increase bacterial loads, mice infected with 5×10^5^ CFU WT were treated with 100 µg of α-IL-17 monoclonal antibody (or rat IgG control antibody) on days 3, 5 and 7 post-infection and bacterial loads were assessed on day 8. Once again there was a modest (2.5-fold) increase in bacterial loads in α-IL-17-treated mice than in control mice (Fig. 7D), though this difference was statistically significant (p = 0.02). To determine whether continued neutralization of IL-17 after the peak of infection during clearance would significantly increase bacterial loads, mice infected with 5×10^5^ CFU WT were treated with 100 µg of α-IL-17 monoclonal antibody (or rat IgG control antibody) on days 3, 6, 9, 12, 15 and 18 post-infection and bacterial loads were assessed on days 14 and 21. As shown in Fig. 7E, there was no significant difference in bacterial loads between treated and control mice at these time points. α-IL-17 antibody-treated mice had reduced neutrophil numbers in the airways compared to control mice (data not shown), demonstrating the efficacy of the antibody treatment. Therefore IL-17 may contribute to protection against *B. pertussis* infection in this model, although apparently not in a dominant manner, and does not seem to contribute to clearance of the infection.

## Discussion

Previously we reported that PT inhibits early (day 1–2) neutrophil influx to the airways in response to *B. pertussis* infection in Balb/c mice [5], [7], [30], through a mechanism involving inhibition of neutrophil-attracting chemokine gene expression [7]. In this report we showed that subsequent to this early inhibitory phase, a second wave of neutrophil recruitment in response to WT *B. pertussis* infection occurs, starting before the peak of infection (day 3–4) and remaining until at least 14 days post-infection, when only low bacterial loads are seen in the airways. This second wave of neutrophil recruitment is stimulated through PT activity by upregulation of cytokines including IL-17, which then upregulates neutrophil-attracting chemokine gene expression. When comparing the infections with the WT and ΔPT strains of *B. pertussis* after inoculation with equivalent doses, there are 2 variables to consider (at least beyond day 1 post-inoculation): (i) the production of PT by the WT strain, and (ii) the higher bacterial load achieved by the WT strain. By inoculating mice with higher doses of ΔPT than WT, we demonstrated that PT, rather than bacterial load, was the more important factor in stimulating these inflammatory responses at the peak of *B. pertussis* infection.

Recent evidence suggests that production of chemokines and subsequent neutrophil recruitment to the airways in response to infection can be induced by a Th17 response, which is associated with the release of IL-17 as well as other pro-inflammatory cytokines. IL-17, in turn, results in the production of chemokines by resident cells of the lung [22], [27], [32], leading to a strong neutrophil recruitment to the airways. The Th17 responses are driven, in part, by the production of IL-6 [24] and are associated with increased release of TNF-α [33]. In our studies, there was significantly greater induction of IL-6 gene expression in response to ΔPT than WT on day 1 post-infection. However, this induction of IL-6 was short-lived and not associated with the production of IL-17 or TNF-α. In response to WT infection, however, there was a strong induction of IL-6 by day 2 post-infection, which preceded the induction of IL-17 and TNF-α, consistent with the established role of IL-6 in Th17 induction [24]. In support of this effect of PT, a recent report demonstrated that PT stimulated the generation of Th17 cells in co-cultured naïve T cells and dendritic cells in an IL-6-dependent manner [34]. Which target cells and signaling pathways PT might affect in vivo to bring about these influences on immune responses remains to be elucidated.

Several reports have shown that infection with *B. pertussis* in both humans and mice induces a Th1 response [18], [35], [36]. However, there are reports indicating that the production of IFN-γ inhibits the induction of IL-17 responses [24], [37] as well as evidence suggesting that IL-17 can inhibit Th1 responses [37]. Nevertheless, a Th1/Th17 response with the production of both Th1 and Th17 cytokines has been described recently [16], [38] and a report by Higgins *et al*. suggested that vaccination of mice with a whole-cell pertussis vaccine leads to the induction of a Th17/Th1 response [16]. In our studies, IFN-γ gene expression was induced strongly in response to the WT strain at the peak of infection, while the induction of IFN-γ in response to ΔPT was relatively low, consistent with the notion that *B. pertussis* infection induces a Th1/Th17 response that is promoted by PT. However, while the induction of the Th17 response was long-lived and gene expression remained up-regulated (several fold) even while the infection was being cleared, the Th1 response was only induced during the peak of infection (days 4 and 7) and gene expression of IFN-γ was reduced to that of control levels by day 14 post-infection. We also detected an increase in IL-10 gene expression near the peak of infection with WT, but not ΔPT, indicating induction of a complex mixture of T cell subtypes during this infection. Overall our data are consistent with the emerging finding that *B. pertussis* infection modulates the immune response towards a Th1/Th17 response.

Our flow cytometry analysis revealed the presence of significant numbers of IL-17^+^ cells in WT-infected mice but not in ΔPT-infected mice, consistent with the gene expression data. We were surprised to find that the IL-17^+^ cells were predominantly in the airways (BAL) rather than the lung tissue. However, this may be a technical artifact due to the relatively lengthy procedure to isolate lung cells during which cytokines may have been released (although in one attempt at in vitro restimulation of lung cells in the presence of Brefeldin A we did not detect any IL-17^+^ cells – data not shown). We were also surprised to find that the IL-17^+^ cells included neutrophils and macrophages as well as T cells. Which of these cells actually secrete the cytokine during infection remains to be determined however, since intracellular staining was used to detect cytokine expression in our analysis.

Several recent studies suggest that toxins released by *Bordetella* species may induce a Th17 response. Adenylate cyclase toxin (ACT) and the type III secretion system may contribute to induction of a Th17 response to *Bordetella* infection [39]. LPS signaling through TLR4 also contributes to Th17 responses in mixed cell culture [40] or after *B. pertussis* infection and pertussis vaccination [16], [20]. We have also found a role for ACT in promoting a Th17 response to *B. pertussis*, and our data suggest that PT and ACT may have a synergistic effect on the induction of Th1 and Th17 cytokines (Andreasen and Carbonetti, manuscript in preparation). Whether the Th17 response plays a role in protection against primary *B. pertussis* infection is still unclear. Higgins *et al*. showed that a possible role for IL-17 in protective immunity to *B. pertussis* was enhancing bacterial killing by macrophages [16]. In our studies, intranasal administration of α-IL-17 antibodies only modestly enhanced peak bacterial growth in the airways and did not delay clearance of the infection, although this treatment did inhibit KC gene expression and neutrophil recruitment to the airways in response to *B. pertussis* infection. We have recently shown that neutrophils are not protective against primary *B. pertussis* infection [8] suggesting that if IL-17 is protective against this infection it must act through another mechanism, such as enhancing macrophage activity. Alternatively, other Th17-associated cytokines, such as IL-22 [21], may be important or may synergize with IL-17 to control this infection. Clearance of primary *B. pertussis* infection involves T cells, since T cell-deficient mice fail to clear the infection [41], but the relative contribution of Th1 and Th17 cells and the mechanisms utilized are still unknown.

Th17 responses have been implicated in the pathogenesis of several chronic immune-mediated pathologies, such as asthma, multiple sclerosis, arthritis and colitis [24], so it is tempting to speculate that this response may also contribute to the pathology of pertussis [1], especially the prolonged cough associated with this disease. We have detected significant IL-17 gene expression and high numbers of neutrophils in the lungs of mice 21 days after *B. pertussis* infection (data not shown - we have not yet assayed later time points), and neutrophilia in the airways has been associated with non-specific symptoms such as cough and bronchial hyperactivity and hypersecretion [22]. Therefore, it is plausible that induction of a Th17 response and subsequent recruitment of large numbers of neutrophils to the airways contributes to the prolonged paroxysmal cough associated with *B. pertussis* infection in humans. Until we have a cough model for this infection we will not be able to test this hypothesis.

## Materials and Methods

### Bacterial Strains

The *B. pertussis* strains used for this study were streptomycin- and nalidixic acid-resistant derivatives of Tohama I and were produced as previously described [5]. The PT-deficient mutant strain (ΔPT) contains an in-frame deletion of PT genes and the wild-type (WT) strain is the parental strain that produces native PT. The PT* strain of *B. pertussis* produces PT with 2 amino acid substitutions in the S1 subunit, rendering the toxin enzymatically inactive and thereby unable to ADP-ribosylate target G_i_ proteins [42]. *B. pertussis* strains were grown on Bordet-Gengou (BG) agar plates containing 10% defibrinated sheep blood and 400 µg/ml of streptomycin.

### Mouse Infection

All mouse procedures were performed in accordance with the Public Health Service Policy on the Humane Care and Use of Laboratory Animals, and with a protocol approved by the University of Maryland, Baltimore Institutional Animal Care and Use Committee. Six-week-old female BALB/c mice (Charles River Laboratories) were used in our studies. Inocula were prepared and mice were inoculated intranasally as previously described [7]. Mice were euthanized by carbon dioxide inhalation at specified time points and the lungs and trachea were removed and homogenized in 2 ml of sterile PBS. Appropriate dilutions were plated on BG blood agar plates with streptomycin and counted after 4 days of incubation at 37°C to determine colony-forming units (CFU) per respiratory tract. A minimum of 3 mice per group was used. A two-tailed *t* test was used for statistical analysis.

### Bronchoalveolar Lavage (BAL)

Mice were euthanized by carbon dioxide inhalation and dissection was performed to expose the trachea and lungs. A 20-gauge blunt-ended needle was inserted into a small incision towards the top of the trachea and tied in place with surgical thread. BAL was performed by flushing the lungs two times with 0.7 ml of sterile PBS. Aliquots of BAL fluid were used for cell counting on a hemocytometer and for cytospin centrifugation. Cytospin centrifugation was performed at 600 rpm for 5 minutes and the slides were stained with modified Wright's stain (Hema 3® Stain Set, Fisher) according to the manufacturer's protocol to identify different cell types. Approximately 100 cells from several microscope fields (5–6) were counted and identified for each sample.

### Lung Perfusion and Cell Preparation

Mice were euthanized by carbon dioxide inhalation and the thoracic cavity was exposed. The aorta was nicked to allow blood to drain and 10 ml of PBS was injected into the right ventricle of the heart, resulting in the removal of blood from the lung tissue. The lung tissue was finely cut with scissors and treated with collagenase type 4/DNAse I for 45 min at 37°C. The digested tissue was passed through a cell strainer (BD Falcon) using the plunger from a 20 ml syringe and the cells were resuspended in sterile PBS.

### RNA Preparation from Lung Tissue

Mice were euthanized by carbon dioxide inhalation and the thoracic cavity was exposed. The two left lobes of the lung were removed, snap frozen in a dry ice/ethanol bath and stored at −80°C until further use. RNA preparation from whole lung tissue was performed using the phenol-chloroform method. Samples were homogenized in 1 ml of RNA Stat-60™ (Tel-Test, Inc.), 200 µl of chloroform was added and the samples were centrifuged at 13,000×*g* for 15 minutes at 4°C. The aqueous phase was transferred to a 1.5 ml tube containing 500 µl of isopropanol and the samples were stored at −20°C overnight. The samples were centrifuged at 13,000×*g* for 15 minutes at 4°C and the RNA pellets were washed twice with 80% ethanol and dried in a DNA Speedvac (Savant) at low temperature for 15 min until the pellet became transparent. The pellet was resuspended in 50 µl of nuclease-free H_2_O and placed in a 65°C water bath for 30 min. Samples were placed on ice and quantification of RNA was performed using a ND-1000 NanoDrop spectrophotometer at 260 nm. Measurements of A_260/280_ were used to determine the purity of the RNA.

### cDNA Synthesis

The synthesis of cDNA was performed with a Reverse Transcription System kit (Promega) according to the manufacturer's protocol using random primers. One µg of RNA was used for each sample. The reaction was incubated at room temperature for 10 min and reverse transcription was performed in a thermal cycler at 42°C for 15 min and 95°C for 5 min. Samples were placed on ice for 5 min to stop the reaction and diluted 1∶10 in nuclease-free H_2_O.

### Real-Time PCR

Real-Time PCR was performed using the SYBR green system (ABI). Primers were generously provided by Dr. Stefanie Vogel (UMB) or designed using Primer Express Software. Primers used were: HPRT forward primer 5′-GCTGACCTGCTGGATTACATTAA-3′; HPRT reverse primer 5′-TGATCATTACAGTAGCTCTTCAGTCTGA-3′; KC forward primer 5′-GCTTGAAGGTGTTGCCCTCA-3′; KC reverse primer 5′-GTGGCTATGACTTCGGTTTGG-3′; LIX forward primer 5′-AGCTGCCCCTTCCTCAGTC-3′; LIX reverse primer 5′-TCCACCTCCAAATTAGCGATCAAT-3′; MIP-2 forward primer 5′-ACCAACCACCAGGCTACAGG-3′; MIP-2 reverse primer 5′-CAGGCATTGACAGCGCAGT-3′; IL-17 forward primer 5′-ACCTCAACCGTTCCACGTCA-3′; IL-17 reverse primer 5′-CAGGGTCTTCATTGCGGTG-3′; IL-6 forward primer 5′-TGTCTATACCACTTCACAAGTCGGAG-3′; IL-6 reverse primer 5′-GCACAACTCTTTTCTCATTTCCAC-3′; TNF-α forward primer 5′-GACCCTCACACTCAGATCATCTTCT-3′; TNF-α reverse primer 5′-CCACTTGGTGGTTTGCTACGA-3′; IFN-γ forward primer 5′-CTGCCACGGCACAGTCATTG-3′; IFN-γ reverse primer 5′-TGCATCCTTTTTCGCCTTGC-3′. A master mix solution was prepared as follows: 12.5 µl of 2X SYBR green master mix, 0.75 µl of the appropriate forward and reverse primers (stock concentration: 10 µM) and 6 µl of H_2_O per sample. Five µl (approximately 30 ng) of cDNA and 20 µl of the master mix solution were added to each well of a 96 well optical reaction plate (Applied Biosystems). All samples, including a H_2_O control, were run in duplicates. The data were analyzed using ΔΔCt calculations and expression of all genes was normalized against the mouse housekeeping gene, HPRT. Results were expressed as fold increase over values from PBS-treated mice.

### Cytokine ELISAs

Mice were euthanized by carbon dioxide inhalation and the thoracic cavity was exposed. The two left lobes of the lung were removed and homogenized in 1.5 ml of Tris-HCl (pH 7.4) containing 1 µg of pepstatin (Roche) and 1 Complete Mini Protease Inhibitor Cocktail Tablet (Roche) per 10 ml solution. The samples were centrifuged at 13,000×g for 5 min and the supernatants were transferred to 1.5 ml eppendorf tubes. This process was repeated two times to remove residual cells. The supernatants were stored at −80°C and ELISA was performed at the UMB Cytokine Core Laboratory.

### Flow Cytometry Analysis

Lung and BAL cells were harvested and prepared as described above. Cells were then surfaced stained with specific antibodies (eBiosciences) for CD11b (Clone M1/70), CD11c (Clone N418), Gr-1 (Clone RB6-8C5), and CD3 (Clone 145-2C11), for 30 min at 4°C, and then washed in staining buffer (2% FBS in PBS). Cells were processed for intracellular staining using Intracellular Staining buffers (eBiosciences) per the manufacturer's instructions and were stained with either an antibody to IL-17 (eBiosciences, Clone eBio17B7) or an isotype control antibody (eBiosciences). Events were collected on a LSRII flow cytometer (Becton Dickinson) driven by FACSDiva software (Becton Dickinson), and analyzed using FlowJo software (Tree Star).

### Intranasal Administration of α-IL-17

Neutralization of IL-17 was performed by intranasal inoculation of 100 µg (in 50 µl PBS) of a rat monoclonal α-IL-17 antibody (R&D Systems). Purified rat IgG (Sigma) was used as a control.

### Statistical Analysis

All statistical analysis was performed using SigmaStat (Systat Software Inc.). Statistical comparisons in gene expression and ELISA experiments were performed using a One-Way ANOVA assay allowing for comparison of three sets of data per time point. A Tukey post-hoc test was used for pairwise comparison of data sets.
